# Editorial: Individual sensitivity to wireless radiation

**DOI:** 10.3389/fpubh.2025.1744897

**Published:** 2025-12-05

**Authors:** Dariusz Leszczynski, Frank de Vocht

**Affiliations:** 1 University of Helsinki, Helsinki, Finland; 2 Centre for Public Health, Population Health Sciences, Bristol Medical School, University of Bristol, Bristol, United Kingdom; 3 National Institute for Health and Care Research (NIHR) Applied Research Collaboration West (ARC West), Bristol, United Kingdom

**Keywords:** electrohypersensitivity, EHS, wireless radiation, proteomics, biomarkers, self-diagnosis

Given the ubiquitous presence of electromagnetic radiation, most notably from wireless technology, there are concerns this could cause an epidemic of various diseases. However, to date such an epidemic that can be clearly linked to wireless radiation exposures, has not materialized. There might be various reasons for this. One plausible explanation being that exposure to electromagnetic radiation does not lead to adverse health effects. It could also be that while wireless radiation results in a variety of biological responses observed *in vitro* or in animals, this only results in adverse effects in a small group of people in the general population who are particularly sensitive [colloquially known as “electro(hyper)sensitives”]. It is well-known and established that different individuals can be differently affected by the same exposure, including those encountered in the environment. There is little reason to assume that this would be different for non-ionizing radiation, including radiation emitted by wireless technology (if indeed there is a causal link with human health). However, such a group of sensitive individuals has not yet been identified in a robust and reproducible manner. There is an ongoing controversy and debate among scientists and the general public about whether the to-date gathered experimental evidence concerning human sensitivity to electromagnetic radiation is of sufficient breadth and quality to prove reliably the existence of individual biological sensitivity to wireless radiation or to disprove this conclusively.

In this Research Topic we collected new research, an account of lived experience, viewpoints and directions for further physiological/psychological research around on ‘Individual Sensitivity to Wireless Radiation'. It includes 7 articles submitted by 21 authors from various European countries and Australia.

The contribution of Ashton provides a first-person lived experience account of his self-diagnosis of electrohypersensitivity (EHS) and journey of living with this condition. He particularly makes the case for further research to try and identify biomarkers that could objectively measure individual sensitivity to electromagnetic radiation and possibly determine/diagnose EHS.

This direction of future research on individual susceptibility is echoed in a contribution by Leszczynski, who argues that individual sensitivity to wireless radiation exists in the same way, or *per analogiam*, with other individual sensitivities to environmental factors. He argues that to-date performed provocation studies are severely biased by the subjectivity of the experimental data, making the results of unreliable and insufficient to claim the lack of causal link of EHS with wireless radiation exposures. In his opinion EHS is a physiological ailment and examining physiology of persons exposed to wireless radiation is the best way to resolve the EHS causality issue. He calls for broader research being a combination of the provocation studies and search for biological markers of response using high-throughput “omics” screening techniques to provide new insights in the understanding individual sensitivity to electromagnetic radiation.

de Vocht and Röösli provide an overview of state of the science around EHS, aimed at separating facts from fiction and beliefs. They discuss why, despite contemporary criticism following consistent null results, randomized provocation trials were considered the most appropriate research methodology to elucidate causal links between EMF exposure and effects in a scientifically robust manner. These have convincingly demonstrated that EMF exposure is not causally related to short-term effects EMF exposure, nor that it can be felt by people with EHS better than chance or the general population. They point out that, if EHS is not of psychosomatic origin, different provocation designs and/or a new biological model should be considered. Although they also see a role for proteomics and other “omics” here, they express some skepticism of these being the panacea for future research.

This in turn aligns with two studies providing evidence of the importance psychosomatic contributions to individual sensitivity and EHS. Bordarie et al. conducted a questionnaire study amongst 100 participants some of which had self-diagnosed with EHS measuring EHS and high sensory processing sensitivity, anxiety and depressive disorders, and absorption, risk perception and avoidance strategies related to EMF, and found an overrepresentation of highly sensitive people within the EHS group. Observed individual differences in terms of anxiety-depressive symptomatology and cognitive strategies suggest avenues for future research and ways to help highly sensitive people, regardless of whether EHS is caused by electromagnetic radiation.

Ledent et al. further conducted two surveys of 97 and 285 participants, respectively, similarly addressing sensitivity to EMFs, modern health worries (MHW), exposure perception, and strategies to limit EMF exposure. They showed how self-reported higher sensitivity to EMFs correlates with greater MHW regarding EMF sources and more strategies to limit EMF exposure. They further show that despite trying to avoid exposures, people who perceive high sensitivity to EMFs remain to feel highly exposed. Ledent et al. suggest exploring new care avenues.

In contrast to psychosomatic contributions, Bijlsma et al. conducted a small pilot double-blind crossover trial of 12 people to explore whether RF exposure impacted on sleep. Interestingly, despite its robust design they only observe that exposure to a 2.45 GHz radiofrequency device (baby monitor) impacts on self-reported sleep in some people under real-world conditions, but not on objective sleep measures actigraphy or polysomnography outcomes, or heart rate variability. They do also report measurable changes to several non-rapid eye movement EEG high frequency measures (Theta, Beta and Gamma waves) but not others (Delta and Alpha waves), which were not observed in rapid eye movement sleep. Given these interesting results they point out the need for further confirmation in large-scale real-world investigations with specified dosimetry.

And finally, the last contribution of this Research Topic explores individual nanoparticle-EMF combined effects in *Drosophila melanogaster*. Bhandari et al. report complex, dose-and time-dependent biological responses to combined 2.4GHz EMF and zinc oxide (ZnO) exposure.

As the articles in the Research Topic highlight, the scientific and societal question on whether some individuals are particularly sensitive to electromagnetic radiation to the point it causes adverse health and psychological effects remains elusive; including whether electromagnetic radiation is to blame at all. Future research will have to consider different approaches, some directions for which being highlighted in the contributing articles, if this conundrum has any realistic hope of being solved.

EHS is not unique because there are numerous health ailments that diagnosis is based solely on patients' subjective descriptions of symptoms. Examples are, the study of pain and, in analogy to EHS that of Myalgic Encephalomyelitis/Chronic Fatigue Syndrome (ME/CFS). Currently efforts are being made to use biomarkers, genetics and epigenetics in the future developing objective tests for pain (1), and recently a blood test for epigenetic markers of ME/CFS has been proposed, albeit based on preliminary data from a small patient group (2). Similar efforts to objectively identify specific biomarkers would be beneficial in the study of EHS as well (3).

Yet, whatever the cause, perceived sensitivity to EMF remains to disproportionally impact the lives of some individuals. In practice it may therefore be prudent for society to try and make amendments, within reason, where family, friends, colleagues or staff identify as suffering from EHS.

Current hypotheses on electromagnetic hypersensitivity constitute vague and sometimes contradictory definitions of outcome (symptoms), exposure metrics (electromagnetic fields), and latencies, and are sometimes constructed *post-hoc*. Symptoms reported by individuals are diverse, subjective, and can be biased. Measuring wrong exposure characteristics or using incorrect latencies between exposure and effects can mask any effects that might be there. The impacts of these factors is compounded by measurement error and misclassification in symptoms and exposure, in part because exposure patterns have changed dramatically over time. These issues make comparisons across different studies difficult, if not impossible. Future research in this area must therefore be based on clearly defined and causal hypotheses that are measurable with respect to exposures and effects. They must also prioritize precise measurement of exposures and outcomes. Only then can results from different studies be compared and the evidence base expanded. The effects of wireless radiation are not fully understood, and caution is needed to avoid unsupported claims based on imprecise data.
